# Identification and characterization of small molecular NPR-B receptor antagonists

**DOI:** 10.1186/2050-6511-16-S1-A70

**Published:** 2015-09-02

**Authors:** Henriette Andresen, Lise Román Moltzau, Steffen Fagerheim, David Gloriam, Trond Bach, Finn Olav Levy

**Affiliations:** 1 Department of Pharmacology, University of Oslo and Oslo University Hospital, Oslo, Norway; 2 School of Pharmacy, University of Oslo, Oslo, Norway; 3 K.G. Jebsen Cardiac Research Centre and Center for Heart Failure Research, University of Oslo, Oslo, Norway; 4 Department of Drug Design and Pharmacology, Faculty of Health and Medical Sciences, University of Copenhagen, Copenhagen, Denmark

## Background

Natriuretic peptides increase in heart failure. Atrial natriuretic peptide (ANP) and brain natriuretic peptide (BNP) activate the natriuretic peptide receptor (NPR)-A and C-type natriuretic peptide (CNP) the NPR-B, causing production of cyclic 3’,5’-guanosine monophosphate (cGMP). Our group has previously shown that CNP potentiates β_1_-adrenoceptor-mediated inotropic response by increasing cAMP levels. This is explained by a crosstalk between cGMP and cAMP, where cGMP produced by NPR-B inhibits phosphodiesterase (PDE) 3 from degrading cAMP. Increased β_1_-adrenoceptor signalling is harmful in heart failure which is the basis for use of β-blockers in heart failure therapy. Further, PDE3 inhibition has been associated with increased mortality in heart failure. Thus, an NPR-B antagonist could eliminate the unwanted effects due to PDE3 inhibition and increased β_1_-adrenoceptor signalling by CNP. There are no selective NPR-B antagonists available, and our aim is to identify and characterize novel non-peptide small molecular selective NPR-B antagonists for potential use in heart failure therapy.

## Methods

Small molecular compounds were screened for activity and selectivity towards NPR-A and NPR-B, using a cGMP assay based on AlphaScreen technology. Based on this technology, we can identify compounds that interfere with BNP- and CNP-stimulated cGMP production through the NPR-A and NPR-B receptor, respectively. Antagonism properties can also be characterized.

## Results and conclusion

Potential NPR-B antagonists were identified by high throughput screening of about 20,000 compounds. Hits were tested against the NPR-A to determine selectivity, and characterized as non-competitive and reversible inhibitors of NPR-B response in HEK293 cells, displaying properties of negative allosteric modulators [1]. Information on structure and activity relationship (SAR), was collected and over 50 new compounds, clustered in five different groups of chemically related compounds, were designed *in silico* and were tested for activity and selectivity towards NPR-A and NPR-B. From this testing, three out of five groups were found interesting. The ligands with the best antagonistic property from each of the 3 groups were used to select 60 more compounds by *in silico* design for further testing.
